# Beyond Readability: Investigating Coherence of Clinical Text for Consumers

**DOI:** 10.2196/jmir.1842

**Published:** 2011-12-02

**Authors:** Catherine Arnott Smith, Scott Hetzel, Prudence Dalrymple, Alla Keselman

**Affiliations:** ^1^School of Library and Information StudiesUniversity of Wisconsin-MadisonMadison, WIUnited States; ^2^Biostatistics and Medical InformaticsSchool of Medicine and Public HealthUniversity of Wisconsin-MadisonMadison, WIUnited States; ^3^Institute for Healthcare InformaticsCollege of Information Science and TechnologyDrexel UniversityPhiladelphia, PAUnited States; ^4^Division of Specialized Information ServicesNational Library of MedicineBethesda, MDUnited States

**Keywords:** Health literacy, comprehension, vocabulary, patients, language tests, retention (psychology)

## Abstract

**Background:**

A basic tenet of consumer health informatics is that understandable health resources empower the public. Text comprehension holds great promise for helping to characterize consumer problems in understanding health texts. The need for efficient ways to assess consumer-oriented health texts and the availability of computationally supported tools led us to explore the effect of various text characteristics on readers’ understanding of health texts, as well as to develop novel approaches to assessing these characteristics.

**Objective:**

The goal of this study was to compare the impact of two different approaches to enhancing readability, and three interventions, on individuals’ comprehension of short, complex passages of health text.

**Methods:**

Participants were 80 university staff, faculty, or students. Each participant was asked to “retell” the content of two health texts: one a clinical trial in the domain of diabetes mellitus, and the other typical Visit Notes. These texts were transformed for the intervention arms of the study. Two interventions provided terminology support via (1) standard dictionary or (2) contextualized vocabulary definitions. The third intervention provided coherence improvement. We assessed participants’ comprehension of the clinical texts through propositional analysis, an open-ended questionnaire, and analysis of the number of errors made.

**Results:**

For the clinical trial text, the effect of text condition was not significant in any of the comparisons, suggesting no differences in recall, despite the varying levels of support (*P* = .84). For the Visit Note, however, the difference in the median total propositions recalled between the Coherent and the (Original + Dictionary) conditions was significant (*P* = .04). This suggests that participants in the Coherent condition recalled more of the original Visit Notes content than did participants in the Original and the Dictionary conditions combined. However, no difference was seen between (Original + Dictionary) and Vocabulary (*P* = .36) nor Coherent and Vocabulary (*P* = .62). No statistically significant effect of any document transformation was found either in the open-ended questionnaire (clinical trial: *P* = .86, Visit Note: *P* = .20) or in the error rate (clinical trial: *P* = .47, Visit Note: *P* = .25). However, post hoc power analysis suggested that increasing the sample size by approximately 6 participants per condition would result in a significant difference for the Visit Note, but not for the clinical trial text.

**Conclusions:**

Statistically, the results of this study attest that improving coherence has a small effect on consumer comprehension of clinical text, but the task is extremely labor intensive and not scalable. Further research is needed using texts from more diverse clinical domains and more heterogeneous participants, including actual patients. Since comprehensibility of clinical text appears difficult to automate, informatics support tools may most productively support the health care professionals tasked with making clinical information understandable to patients.

## Introduction

A basic tenet of consumer health informatics is that understandable health resources empower the public by increasing knowledge and improving decision making [1]. Research indicates that most laypeople have difficulty comprehending medical documents, especially those that describe complex information pertaining to clinical research (for example, [2]). For example, numerous studies suggest that most patients, especially those with lower income levels and literacy skills, have difficulty reading and comprehending informed consent documents [3]. Poor understanding of health information thus has an impact on public health. This has prompted many research efforts to close the gap between the difficulty level of documents and readers’ literacy by improving the readability of health-related materials.

*Readability* itself is a concept drawn from kindergarten to grade 12 education, in which field research findings suggest that readers’ ability to comprehend a passage decreases as the number of “difficult” words (that is, words unfamiliar to the average reader) increases. Typically, readability measures are derived from sentence and word length. Substituting simpler, more familiar vocabulary improves readability in texts whose subject matter addresses general knowledge domains. (For a comprehensive review, see [4]).

Attempts to lower the readability level of health materials, usually to grades 7–9, have successfully employed these techniques, but health information presents additional challenges. Health information not only contains unfamiliar and difficult words, but also abounds with complex concepts such as those related to physiology and pharmacology. In addition, understanding health materials requires readers to make inferences that access a body of specialized knowledge supporting the information. Experts possess this specialized body of knowledge and so are able to make inferences, but even highly educated nonspecialists may not be able to make those inferences because they lack the necessary training and experience.

### Literature Review

#### Patients and Clinical Language

Medical terminology has long been recognized as a specialized language that is acquired through education and clinical practice [5]. For decades, medical terminology has been cited by physicians as a significant concern about patients’ possible misinterpretation of medical record content [6-10]. Much of this literature derives from early controversies over patient access to records—first, in psychiatry, proposed initially as an entirely theoretical construct by Westin [11], and then, in the early years of the British Access to Health Records Act, which in 1990 first gave British patients access to their medical information “held in manual form” [8].

Surprisingly, given the early concerns expressed about patient understanding of medical terminology, few studies published since the mid-1990s have examined the problem. Only Tomkins et al [12] examined patients’ comprehension of physician letters. Far more common is the assertion that medical terminology is a nail, and customized patient education materials the appropriate hammer. For example, Nijland et al [13] found that terminology was a barrier to usability in patient self-care applications, concluding that “Self-care support applications should match the vocabulary of the users and the language of the medical systems.”

The medical terminology problem is compounded by the consumer health vocabulary problem: that the everyday language used by consumers to describe diseases and treatments is a hybrid of specialized terms and common words that are part of general spoken vocabularies (see Keselman et al [14] for a discussion of research in this area to date). Consumer terms are also characterized by levels of granularity and specificity different from their medical counterparts. For example, anatomical words such as “blood” or “brain” usually suffice for lay discussion of physiology or pathology, while the specialist requires terms that describe much smaller, more specific aspects of the blood or the brain. For these reasons, any approach to vocabulary simplification is challenging, particularly for analysis by reading level. Words that contain many syllables, such as “hospitalization,” contribute to a higher calculated reading level for that document, yet the term hospitalization is easily understood by laypeople who know what a hospital is; conversely, short and simple words such as “gene” or “immune” are terms referencing complex entities and processes.

Carefully designed entry vocabularies may be able to serve as bridges between terms and concepts used by different user communities. This idea was the impetus for development of the Unified Medical Language System, which focuses on the numerous sublanguages of health care. The idea that consumers constitute a user community of their own, however, is more recent (see [15] for a review), and details of how such an entry vocabulary can be used in real-world implementations are lacking. Zeng and Tse [16] argued in opposition to Patrick et al [17] that simply providing users with a list of medical terms or a dictionary would not solve the terminology problem for informatics applications. More recently, Leroy and Miller [18] found some evidence supporting Zeng and Tse. This reading comprehension study investigated the effect of automatically generated health topics overviews (HTOs). These HTOs, described as functioning “much as a table of contents,” were overviews, not dictionaries, but like dictionaries were intended to function as information assists. Leroy and Miller found that “vulnerable” consumers—those identified as having low health literacy or high stress—were found to rely on the HTO even more than they did on the text that the HTO summarized—so much so that vulnerable consumers performed more poorly when an HTO was not available.

Dictionaries can be considered a good starting point for language bridges, since they contain definitions presumed to be standard and thus common across communities. However, the sociocultural dimensions of term variability require more depth and breadth of expression than any dictionary or glossary can effectively provide without a great deal of customization; Velardi and colleagues have commented on the resource intensiveness of the interactive glossary process itself [19]. Indeed, the literature of medical informatics is largely silent on this question, since the usefulness of dictionaries or glossaries as terminology support in health informatics is an untested assumption, with one exception: Diefenbach and Butz [20] constructed a virtual health center for use in educating patients with prostate cancer. The library “room” in this health center used a glossary in which some medical terms were hyperlinked to short definitions. A focus group of patients and spouses identified the glossary as helpful.

#### Coherence

Text coherence, a concept from the fields of cognitive psychology and education, refers to the connectedness of ideas in a text, which affects comprehension [21]. A distinction is usually made between *local* and *global* coherence. Local coherence refers to the explicit connection between adjacent clauses and sentences, also referred to as *cohesion*; global coherence refers to the logical organization in which macro-level ideas are presented. [22].Text coherence is the function of both text and reader; an identical text may be perceived as either well or poorly connected, depending on the reader’s background knowledge. Extracting meaning from text always requires some inferences, and it is the background knowledge that determines whether the needed inferences are trivial or insurmountable.

When discussed as a feature of a text, coherence usually applies to its “average” reader, or target audience. When it comes to comprehending medical information, laypeople lack the in-depth knowledge of the domain, an expertise that is characteristic of the professional who wrote those texts. Medical documents such as those contained in electronic and personal health records, informed consent forms, and medication instructions are likely to refer to concepts and make connections with which readers may not be familiar.

To support consumer comprehension, we must bridge the gap that exists between writers’ and readers’ knowledge: between the elaborate background expertise of the writer that serves as a basis for assumptions in the text, and the more modest background familiarity of the reader. In other words, we need to make the text more “coherent,” ensuring that its ideas are well connected not only with each other, but with the likely background knowledge of the intended reader; that the number of inferences, or mental leaps, required of the reader moving from one idea to the next is not excessive; and that these inferences are easy to make.

Consider the following statement: “After Jennifer mentioned that her daughter was ‘always thirsty,’ the doctor asked if she had recently lost weight.” A reader with some knowledge about type 1 diabetes will know that thirst and weight loss are both symptoms of diabetes. Such a knowledgeable reader will infer the connection between the two clauses of the sentence, will understand that the word “she” refers to the daughter rather than the mother, and may even anticipate the doctor’s next questions. The reader without prior knowledge of diabetes, however, will not be able to make the connection.

Coherent texts ensure that less effort is required for the reader to transition from clause to clause, extracting meaning and building a mental representation of the text. In comprehension research, text memory and mental representations are typically measured in terms of *propositions*. A proposition is the smallest meaningful unit of thought, often consisting of two concepts and a relationship that connects them (antiobiotic_TREATS_infection), or a concept and a modifier (infection_IS_bacterial). Propositions typically correspond to sentence clauses. Not every proposition of the original text is encoded and remembered [23]: concepts and relationships that are connected to the reader’s prior knowledge are more likely to be retained.

Reduced comprehension effort is not necessarily always better for all readers. In fact, studies suggest that when readers with strong background read less coherent texts, they are forced into deeper processing, and actually learn more [21,24]. For less knowledgeable readers, however, lack of coherence in the text is detrimental to comprehension and learning. As they lack background knowledge concepts to which they can relate the text, they remember little, and build representations characterized by omissions and errors [25]. Laypeople reading medical documents are likely to fall into the category of less knowledgeable readers, those whose comprehension would benefit from more coherent texts.

Little is known about the coherence of standard medical documents, because research into the comprehensibility of these materials has typically focused on readability. As noted above, however, readability does not ensure coherence. Local coherence is likely to be compromised by the unfamiliar concepts and relationships between them—as in the example given above regarding thirst and weight loss—as well as by general writing style issues, neither specific nor limited to the health domain. Global coherence, additionally, is likely to be compromised if the overall structure of the documents reflects health professionals’ rather than lay conception of health and disease.

Studies in cognitive psychology suggest that rewriting texts using explicit coherence principles, rather than writers’ intuition, leads to improved comprehension for less knowledgeable readers. McNamara et al [26] analyzed 12 available studies that revised texts to change their coherence (which these authors refer to as cohesion). Principles for improving local coherence typically involve strategies such as the addition of argument overlap (making each sentence repeat the linking word from a previous sentence), the use of sentence connectives, and the rearrangement of clauses so that sentences repeat old ideas before introducing new ones. Improving global coherence involves introducing background concepts; making important references explicit; explaining causal connections between events; adding headers and topic sentences; and clearly linking subtopics to the main topic [21,25,27,28].

Although published studies describe many strategies for improving text coherence, they do not provide specific guidance for choosing among them. Most studies use a combination of techniques, directed at improving both local and global coherence. Vidal-Abarca et al [27] explicitly compared the effect of local versus global coherence improvements in a history text on the Russian Revolution, and concluded that global, but not local, coherence improvements led to deeper comprehension, as measured by the ability to answer inference questions and focus on main ideas during recall. Vidal-Abarca and colleagues also concluded that the strongest benefits for comprehension were produced by a version with both local and global coherence revisions. To the best of our knowledge, no studies have compared the impact of local versus global comprehension revisions as a function of text difficulty. It is reasonable to expect that global coherence revisions, which target gaps in knowledge, are more essential for texts in knowledge-rich domains, such as history or medicine, than for domains with weaker ties to specialized subject knowledge, such as fiction. At the same time, one should keep in mind that conceptual complexity (and thus global coherence) is not purely a characteristic of a text, but of a match between the text, the knowledge and intention of its authors, and its reader. This makes global coherence editing more art than science, compared with local coherence editing.

While the cognitive psychology literature outlines rather specific principles for improving text coherence, professional writers of health education brochures have a wide range of notions about what it means for the text to be coherent and how coherence can be achieved (Kools et al [29].) Certain specific principles of coherence were overlooked by these writers—for example, the use of sentence connectives to clarify relationships, especially causal relationships, between concepts; and the correct use of word order, to make clear that new information is related to information previously given to the reader. Extending the focus of consumer health comprehension research beyond readability to include coherence is likely to lead to insights about ways to support patients’ understanding of medical documents.

### Study Goals

The need for efficient ways to assess consumer-oriented health texts, and the availability of computationally supported tools to accomplish these tasks, led us to explore the effect of various text characteristics on readers’ understanding of health texts, as well as to develop novel approaches to assessing these characteristics. We were particularly interested in coherence and the complexity of health-specific vocabulary. To explore these issues, we conducted an exploratory study to compare two approaches to improving the readability of health materials. One approach focuses on identifying and explaining difficult words; the other focuses on identifying logical gaps and providing additional texts to facilitate inference, thereby increasing coherence.

The goal of this study was to compare the impact of three interventions on individuals’ comprehension of short, complex passages of health text. Two interventions provided terminology support via (1) standard dictionary, or (2) contextualized vocabulary definitions developed specifically for the study. The third intervention provided coherence improvement. The Methods section describes these interventions in detail. We tested the following 4 hypotheses.

H1: Readers’ comprehension of a text enhanced by providing standard, off-the-shelf dictionary definitions (hereafter referred to as the Dictionary condition) will be equivalent to their comprehension of the original text (hereafter referred to as the Original condition).

H2: Readers’ comprehension of a vocabulary-enhanced text (hereafter referred to as the Vocabulary condition) will be significantly greater than in the Original and Dictionary conditions combined.

H3: Readers’ comprehension of a text with improved coherence (hereafter referred to as the Coherent condition) will be significantly greater than in the Original plus Dictionary conditions.

H4: Readers’ comprehension of the Coherent condition will be significantly greater than in the Vocabulary condition.

## Methods

### Participants

A total of 80 people associated with the University of Wisconsin-Madison as staff, faculty, or students participated in the study. Participants were recruited in two cohorts. The first cohort of 40 participants consisted of mixed faculty, staff, graduate, and undergraduate students recruited via campus fliers and newspaper advertising. After we determined that the initial sample size was insufficient to capture the effects of the interventions, we recruited a second cohort, consisting entirely of graduate students in library and information studies, from the University of Wisconsin-Madison School of Library and Information Studies, via an in-class announcement. All participants completed the tasks individually and received $25 bookstore gift cards for participating. The study was approved by the Social Sciences Institutional Review Board of the University of Wisconsin-Madison on February 23, 2007.

All participants completed an anonymous demographic questionnaire to report their gender, age, racial/ethnic characteristics, educational level, and work experience. Participants also self-rated their biomedical understanding on a scale from 1 (“I rarely read texts on biomedical topics”) through 4 (“I read and understand general medical articles”) and their knowledge about diabetes mellitus on a scale from 1 (“very little”) to 5 (“a good deal”).

Table 2 (see the Results section) shows the characteristics of the sample obtained from this questionnaire.

### Document Types

#### Clinical Trial

The first document type (see Textbox 1) was a description of a clinical trial entitled “Non invasive assessment of liver glycogen kinetics and ATP synthesis in type 1 diabetics”, adapted from ClinicalTrials.gov (database trial identification number NCT00481598), the largest existing registry of clinical trials, maintained by the National Library of Medicine. This trial was selected because it concerned diabetes mellitus, a common diagnosis, and because the documentation of the trial included a description of the study’s purpose. This made it ideal for a study assessing consumer comprehension of text, as opposed to other dimensions of health literacy, such as understanding of tabular data, or numeracy in general. In fact, McCray and Ide [30] wrote early in ClinicalTrials.gov’s history that one motivation for creating this website was the desire to make clinical trial information “available to individuals with serious or life-threatening diseases and conditions, *to other members of the public* [our emphasis], to health care providers, and to researchers” and available “in a form that can be readily understood by members of the public.” Leroy and colleagues similarly chose a clinical trial document for a readability study because it represents “the most difficult language...that consumers will encounter and are expected to understand, that is, [a document] meant for them.” [31]

#### Visit Notes

The second document type was a sample cardiology office Visit Notes document (Textbox 2) obtained from an online collection of sample medical transcripts at MedicalTranscriptionSamples.com. The site is a reference resource for medical transcriptionist training. The Visit Notes document was selected because of its focus on heart disease, a common consumer health concern. The document included the following sections: (1) history of present illness, (2) physical examination, (3) medications, (4) diagnoses, and (5) plan. A nurse practitioner reviewed the document and found it representative of office Visit Notes.

**Clinical Trial Document**

NCT00481598 Non Invasive Assessment of Liver Glycogen Kinetics in Type1 Diabetics

Patients with Type 1 diabetes suffer from impaired postprandial hepatic glycogen storage and breakdown, if they are under poor glycaemic control. Poor glycogen storage in the liver puts these patients at risk of fasting hypoglycemia. Amelioration of glycaemic control could improve these abnormalities and thereby reduce the risk of hypoglycemia in these patients. The “gold standard” technique for the assessment of hepatic glycogen metabolism in humans, 13 C magnetic resonance spectroscopy (13C-MRS), is expensive and limited to a few centers worldwide. Aim 1 of our project is to establish a new assessment method for glycogen metabolism. This new method is based on oral administration of 2H2O and acetaminophen.

**Visit Notes document**

History of Present Illness:

This 66-year-old white male was seen in my office on Month DD, YYYY. Patient was recently discharged from Doctors Hospital at Parkway after he was treated for pneumonia. Patient continues to have severe orthopnea, paroxysmal nocturnal dyspnea, cough with greenish expectoration. His exercise tolerance is about two to three yards for shortness of breath. The patient stopped taking Coumadin for reasons not very clear to him. He was documented to have recent atrial fibrillation. Patient has longstanding history of ischemic heart disease, end-stage LV systolic dysfunction, and is status post ICD implantation. Fasting blood sugar this morning is 130.

Physical Examination:

VITAL SIGNS: Blood pressure is 120/60. Respirations 18 per minute. Heart rate 75-85 beats per minute, irregular. Weight 207 pounds.

HEENT: Head normocephalic. Eyes, no evidence of anemia or jaundice. Oral hygiene is good.

NECK: Supple. JVP is flat. Carotid upstroke is good.

LUNGS: Severe inspiratory and expiratory wheezing heard throughout the lung fields. Fine crepitations heard at the base of the lungs on both sides.

CARDIOVASCULAR: PMI felt in fifth left intercostal space 0.5-inch lateral to midclavicular line. First and second heart sounds are normal in character. There is a II/VI systolic murmur best heard at the apex.

ABDOMEN: Soft. There is no hepatosplenomegaly.

EXTREMITIES: Patient has 1+ pedal edema.

Medications:

1. Ambien 10 mg at bedtime p.r.n.

2. Coumadin 7.5 mg daily.

3. Diovan 320 mg daily.

4. Lantus insulin 50 units in the morning.

5. Lasix 80 mg daily.

6. Novolin R p.r.n.

7. Toprol XL 100 mg daily.

8. Flovent 100 mcg twice a day.

Diagnosis:

1. Atherosclerotic coronary vascular disease with old myocardial infarction.

2. Moderate to severe LV systolic dysfunction.

3. Diabetes mellitus.

4. Diabetic nephropathy and renal failure.

5. Status post ICD implantation.

6. New onset of atrial fibrillation.

7. Chronic Coumadin therapy.

Plan:

1. Continue present therapy.

2. Patient will be seen again in my office in four weeks.

### Study Conditions

We transformed the original documents three times to create the study conditions: health dictionary support (for the Dictionary condition), contextualized vocabulary support (for the Vocabulary condition), and coherence enhancement (for the Coherent condition). Each transformation is described below. For a summary comparison of characteristics of the original and transformed texts, see Table 1 in the Methods section, below.

#### Health Dictionary Support Transformation (Dictionary Condition)

We applied the predictive health term difficulty algorithm created by Zeng et al [5] to each document in order to identify terms unlikely to be familiar to consumers. Additionally, three nonclinician researchers independently extracted all potentially difficult health-related terms and expressions from the texts, adding them to the list of terms needing additional explanation. Finally, a nurse practitioner identified any remaining terms that were potentially problematic. These terms were selected for dictionary support.

**Figure 1:**
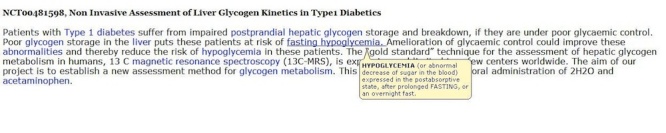
Text selection with example of dictionary support.

“Difficult” terms were highlighted in blue in the text of the Dictionary condition. Terms in this condition had definitions provided in pop-up balloons activated by mousing over the text presented on a computer screen (see Figure 1). We culled definitions of terms from readily available Internet dictionary sources, such as Merriam-Webster’s Medical Dictionary and others identified by Google’s “define” function.

#### Contextualized Vocabulary Support Transformation (Vocabulary Condition)

**Figure 2:**
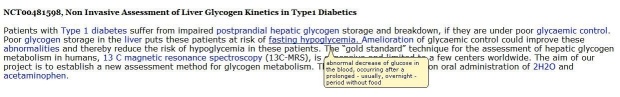
Text example with contextualized vocabulary support.

This was similar to the transformation undertaken for the Dictionary condition described above, but in the Vocabulary condition, term definitions appearing in the pop-up balloons were edited by the nurse practitioner to specifically apply to the terms’ contextual usage in the documents (see Figure 2).

#### Coherence Enhancement (Coherent Condition)

This condition was developed in collaboration with the nurse practitioner, based on the principles outlined in the literature review above. We attempted to increase document coherence both at the local level (that is, between adjacent sentences) and at the global level (that is, across all sentences of the document), without altering the texts’ graded readability level as measured by the Flesch-Kincaid formula [32]. Given the very different structures and the different intended original audiences for the two documents, we used two different procedures for improving their coherence.

While we attempted to target both local and global coherence, local coherence was not applicable to the sections of the Visit Notes that contained numbered items rather than sentences. This is because local coherence deals with sentence overlaps, often mentioning a concept from the previous sentence at the beginning of a new one; this makes it clear and unambiguous what various pronouns refer to (eg, does “it” refer to the heart or the procedure performed on it?). When text consists largely of bulleted or numbered lists, it is hard to do this kind of local coherence correction. For example, in a medications list made up of numbered sentence fragments, concepts mentioned in new sentences cannot be clearly linked to earlier sentences.

Global coherence, conversely, presents a contextual issue rather than a compositional one. We felt that coherence gaps in both documents had to do with the lay readers’ insufficient background knowledge, leading to difficulty making inferences. In addition, potential coherence-related difficulties with the Visit Notes could be related to the topical organization and section and subsection headers in the document, a structure highly conventional and likely very familiar to medical professional authors and readers, but not to laypeople. The procedures by which we improved the coherence of the texts are described in detail below.

### Clinical Trial Document Type

**Figure 3:**
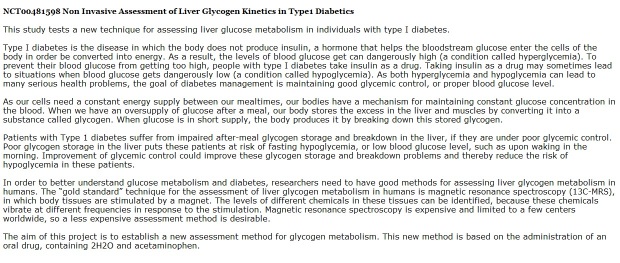
Clinical trial document with coherence enhancement.

We first segmented this text into units of analysis, usually complete sentences. In some cases, complex sentences were divided into propositions, keeping intact phrases beginning with words such as “therefore” or “because.” Next, we identified coherence gaps, defined as places where an inference was needed to comprehend each sentence on the basis of preceding sentences. Information was then added to the text, either by supplementing existing sentences or by adding new sentences that contained contextualized explanations. Examples of such added information include a missing background concept—for example, an explanation of the dangers of hypoglycemia—or the rationale behind the assessment procedure—for example, explaining the need to have good methods for measuring liver glycogen metabolism. Additionally, to make the clinical trial’s research objectives more obvious, information about the purpose of the trial was rearranged from its original location so that it appeared in the opening sentence of the transformed document. Finally, to ensure local coherence, we checked the final text to ensure that the referents of pronouns were explicit. The coherence-transformed clinical trial text appears in Figure 3.

### Visit Notes Document

The revision of this document involved a macrostructure analysis, performed by the nurse practitioner. This involved analyzing the relationship between sections of the document and the logic of the thematic organization of information within these sections. The nurse practitioner noted that grouping of diagnoses and complaints in the History of Present Illness and Diagnosis sections of the original document lacked a particular order. These complaints were accordingly regrouped into *heart-related*, *diabetes-related*, and related to *breathing difficulties*. “Chief complaint” was added to the breathing difficulties subheading. Just as the research objectives of the clinical trial were made more prominent in the transformed text, so for the Visit Note, medical concepts were explained in the body of the document. For example, in the Physical Examination section, test results were explained and interpreted (eg, by placement within or outside the normal range). In the Medications section, medications were regrouped by function; specific functions (eg, breathing problems; heart function and blood pressure) were explained, as were methods of action of individual medications.

Once we completed the coherence editing, we analyzed the text for readability level according to the Flesch-Kincaid Grade Level formula [32]. Based on these findings, adjustments were made to the Coherent condition of the clinical trial document, to ensure that its readability level remained comparable with that of the original text. In the case of the Visit Notes, the readability score for the coherent version was significantly higher than that of the original (see Table 1). As was noted early by Chapman et al [33], calculation of reading level using Flesch-Kincaid can be problematic for clinical text, because this formula relies partly on sentence length to establish difficulty, and medical documentation can be written in very short sentences. In our Visit Note, many sections of the original text were not written in complete sentences, resulting, in our estimation, in a deceptively low grade. In this case, we decided not to attempt matching readability levels, deeming that the Flesch-Kinkaid formula underestimated the difficulty of the original.

**Table 1:** Text characteristics of documents

Document type and condition		Number of words	Number of vocabulary definitions	Number of sentences	Grade level (Flesch-Kincaid)
**Clinical trial**					
	Original	108	NA^a^	6	14.1
	Dictionary	^b^	12	^b^	^b^
	Vocabulary	^b^	12	^b^	
	Coherent	394	NA^a^	18	13.8
**Visit Notes**					
	Original	326		43	9.5
	Dictionary	^b^		^b^	
	Vocabulary	^b^	12	^b^	
	Coherent	1219		66	11.3

^a^ Not applicable.

^b^ Dictionary- and Vocabulary-enhanced versions had the same number of words and same Flesch-Kincaid Grade Readability Level and sentences as the original versions.

### Procedure

Study participants worked on individual computers; a research assistant served as proctor, observing at all times to ensure that work was done individually. Participants were randomly assigned to one of the four conditions (Original, Dictionary, Vocabulary, or Coherent). It was explained to all participants that some of them would see balloon features in their documents, and that they should feel free to take advantage of these features.

The order of presentation of the clinical trial and Visit Notes documents was randomized among participants. After completing the anonymous demographic questionnaire, participants read their first document on the computer screen. After a waiting period of 10 minutes, participants wrote their recollection of the text they read in this document using Microsoft Word. They were instructed to retell the document they had just read as if they were making the information available to a person who had never seen it before. In addition, participants in the second, but not the first, cohort answered an open-ended questionnaire about the text they read (see Textbox 3). This procedure was then repeated for each participant’s second document.

Observation during the session indicated that participants did indeed invoke the balloon features. Participants were allowed to take as long as they required to “retell” each text; the modal time to completion was 20 minutes in both cohorts. The time period was selected to be sufficient for all participants to complete the task without pressure, regardless of the length of the stimulus text. All participants were able to finish their work before the time elapsed.

**Open-Ended Questionnaire for Clinical Trial Text.**

1. Who is being recruited for the study described in this paragraph?

2. This paragraph mentions measuring something. What is the thing that is being measured?

3. Why is it important to measure this thing?

4. Many health problems are associated with diabetes. Which particular health problem is the main focus of this text?

5. What is the innovation of the research described in this text?

### Coding and Statistical Analysis

There were three outcomes of interest: (1) number of propositions recalled, (2) open-ended questionnaire score, and (3) number of errors made by participants. These were collected over the four study conditions for each of the two document types. We assessed the effect of the conditions on the outcomes separately for each document type.

#### Demographic Questionnaire Analysis

Demographic variables were summarized by frequency and percentage or median and interquartile range (IQR) based on the distribution type of each variable. We compared demographic factor variables between the four groups with Fisher exact tests. We compared demographic score variables between the four groups with Kruskal-Wallis tests. The Kruskal-Wallis test was used because it is the nonparametric test for comparing more than two groups. All demographic comparisons were insignificant so no pairwise comparisons were made.

#### Propositional Analysis

We followed the standard procedure of segmenting original versions of each text into propositions, or basic units of analysis corresponding to two concepts connected by a relationship (eg, [antigen] attacks [immune system]) or a concept with a modifier (eg, severe [pain]) [34]. Disagreements about whether a particular statement constituted a proposition were resolved via discussions among three of the authors (AK, CAS, and PWD). Scoring was based on participants’ recall of the propositions of the original texts. Lists of propositions found in the original texts were used as scoring sheets against which to analyze participants’ recall.

Each transcript was scored to indicate the presence or absence of the original text’s proposition in the retelling. The coding guide was developed through discussions, using a pilot (training) dataset. We obtained the pilot retellings from the our colleagues and family members with demographics similar to the participants’. Two raters (AK and CAS) scored three randomly selected pilot retellings of each document. The analysis of interrater reliability yielded kappa coefficients of .73 (substantial agreement), .8 (almost perfect agreement), and .83 (almost perfect agreement) for the Visit Notes and .71 (substantial agreement), .76 (substantial agreement), and .8 (almost perfect agreement) for the clinical trial. Disagreements were resolved via discussions, following which AK and CAS each scored half of the protocols. The transcripts were scored in random order and the scorer was blind to the condition being scored [35].

#### Open-Ended Questionnaire

We administered an open-ended questionnaire to each participant in Cohort 2, one questionnaire for each document type for a total of two questionnaires per participant. (For an example of the clinical trial document’s open-ended questionnaire, see Textbox 3 above). Authors CAS and AK jointly coded all the questionnaires and resolved disagreements through mutual discussion. The clinical trial questionnaire was scored by assigning each answer a score of 0, 1, or 2, reflecting the accuracy and completeness of participants’ answers; for the Visit Notes questionnaire, since answers reflected retention and understanding of much more granular information, a point was awarded for each medication, diagnosis, etc. recalled correctly by the participant.

### Statistical Analysis

Similar statistical analysis was performed for (1) the number of propositions recalled, (2) open-ended questionnaire score, and (3) number of errors. For each variable, initially, a Kruskal-Wallis test was performed to test for differences in the outcomes based on the four study conditions. In those circumstances in which the Kruskal-Wallis test was insignificant, the initial hypothesis that there would be no difference between the Original and Dictionary conditions was tested with a Wilcoxon rank sum test. If this test was also insignificant, then these two groups were combined and Kruskal-Wallis analysis was rerun comparing the three condition groups as follows: (1) Original + Dictionary, (2) Vocabulary, and (3) Coherent. If this Kruskal-Wallis test was significant, then pairwise Wilcoxon rank sum tests were conducted with Holm adjusted *P* values for multiple comparisons. All comparisons were conducted at an alpha level of .05.

In addition, post hoc power analysis was done for comparison of the Visit Notes total open-ended questionnaire scores among the four conditions. This analysis was done for the Visit Notes, but not for the clinical trial, because for the Visit Notes, the distribution of the medians for the four conditions showed a steady trend in the expected direction.

## Results

Table 2 shows results of the experiment and characteristics of the participants

**Table 2:** Characteristics of participants

Variable		Intervention group				*P* value
		Original	Dictionary	Vocabulary	Coherent	
**Gender, n (%)**						
	Female	17 (85)	15 (75)	17 (85)	15 (75)	.59
	Male	3 (15)	5 (25)	3 (15)	5 (25)	
**Age (years), n (%)**						.48
	<30	15 (79)	14 (70)	12 (60)	15 (75)	
	30–39	2 (11)	4 (20)	4 (20)	0 (0)	
	40–49	1 (5)	1 (5)	2 (10)	4 (40)	
	50–65	1 (5)	1 (5)	1 (5)	1 (5)	
	>65	0 (0)	0 (0)	1 (5)	0 (0)	
**Education level attained, n (%)**						.91
	High school	3 (15)	2 (10)	3 (15)	2 (10)	
	College degree	12 (60)	12 (60)	10 (50)	12 (60)	
	Master’s	5 (25)	5 (25)	7 (35)	4 (20)	
	>Master’s	0 (0)	1 (5)	0 (0)	2 (10)	
**Degree typea, n (%)**						1.00
	Health-related	1 (5)	1 (6)	1 (6)	1 (5)	
	Nonhealth-related	18 (95)	17 (94)	17 (94)	19 (95)	
**Biomedical knowledge**						
	Median (IQR)^b^	1.5 (1.0–3.0)	2.0 (1.0–2.0)	2.0 (1.0–2.0)	2.0 (2.0–3.0)	.15
**Diabetic knowledge**						
	Median (IQR)	2.0 (1.0–3.0)	2.0 (1.8–3.0)	2.0 (2.0–3.3)	3.0 (2.0–3.3)	.72

^a^ Of highest earned degree.

^a^ Interquartile range.

### Number of Original Text Propositions Recalled

The effect of the version on the number of the original text propositions recalled was assessed separately for each document type, clinical trial and Visit Notes alike. Both document types showed insignificant differences between the Original and Dictionary conditions (*P* = .65, *P* = .48, respectively). The two conditions were combined for the subsequent analysis.

For the clinical trial text, the effect of the condition was not significant in any of the comparisons, suggesting no differences in recall, despite the varying levels of support (*P* = .84). For the Visit Note, however, we found a significant difference in the median total propositions recalled between the Coherent and the (Original + Dictionary) conditions (*P* = .04). This suggests that participants in the Coherent condition recalled more of the original Visit Notes content than did participants in the Original and the Dictionary conditions combined. No comparisons involving the Vocabulary condition were significant. Median, IQR, and range for the number of propositions recalled for each document type are presented in Table 3.

**Table 3:** Total propositions recalled

Document type and condition		n	Median	IQR^a^	Range	Contrast	*P* value
**Clinical trial**							
	Original	20	8.5	6.75–12.25	4–21	O^b^ vs D^c^	.63
	Dictionary	20	9.0	7.0–13.25	5–23		
	Combined (O^b^ + D^c^)	40	9.0	7.0–13.0	4–23	Kruskal-Wallis	.84
	Vocabulary	20	10.0	6.75–12.25	2–18		
	Coherent	20	10.5	7.75–13.5	4–18		
**Visit Notes**							
	Original	20	17.5	14.0–21.25	9–39	O^b^ vs D^c^	.48
	Dictionary	20	20.0	16.5–23.25	4–41		
	Combined (O^b^ +D^c^)	40	19.0	15.0–22.0	4–41	O^b^ + D^c^ vs V^d^	.36^e^
	Vocabulary	20	22.5	15.75–32.75	5–50	V^d^ vs C^f^	.62^e^
	Coherent	20	25.5	20.5–33.25	13–41	O^b^ + D^c^ vs C^f^	.04^e^

^a^ Interquartile range.

^b^ Original.

^c^ Dictionary.

^d^ Vocabulary.

^e^ Holm adjusted *P* values for multiple comparisons.

^f^ Coherent.

### Open-Ended Questionnaire Scores

This comparison involved the effect of the conditions on the open-ended questionnaire scores. For both text types, the initial Kruskal-Wallis comparison of the Original and Dictionary conditions was insignificant (clinical trial: *P* = .70, Visit Note: *P* = .36), so the two conditions were combined. The analysis found no significant effect of the text version in any of the clinical trial comparisons (*P* = .86). The effect of the text version for the Visit Notes also did not reach significance (*P* = .20). Median, IQR, and range for the number of main ideas for each document type are presented in Table 4.

**Table 4:** Open-ended questionnaire scores

Document type and condition		N	Median	IQR^a^	Range	Contrast	*P* value
**Clinical trial**							
	Original	20	6.0	3.25–8.0	1–10	O^b^ vs D^c^	.70
	Dictionary	20	6.0	4.5–8.0	3–10		
	Combined (O^b^ + D^c^)	40	6.0	3.75–8.0	1–10	Kruskal-Wallis	.86
	Vocabulary	20	5.5	5.0–7.0	3–8		
	Coherent	20	6.0	4.25–6.0	3–9		
**Visit Notes**							
	Original	20	11	10.25–14.25	7–20	O^b^ vs D^c^	.36
	Dictionary	20	13.5	12.25–15.5	9–16		
	Combined (O^b^ + D^c^)	40	12.5	10.75–15.25	7–20	Kruskal-Wallis	.20
	Vocabulary	20	14.0	13.0–18.75	7–20		
	Coherent	20	15.0	14.25–15.0	10-18		

^a^ Interquartile range.

^b^ Original.

^c^ Dictionary.

Because the open-ended questionnaire was added to the study after the first half of the participants completed the study, the data sample was small, consisting of 10 participants per condition. We performed a post hoc power analysis for the test of differences between the four treatment conditions for Visit Notes open-ended questionnaire scores. With the assumption of normal data with means 11, 13.5, 14, and 15, which were the median values seen in the actual data, and overall standard deviation of 3.4, the post hoc power analysis indicated that we had only 57% power to find a difference with 10 participants per condition. To achieve adequate 80% power to detect a difference, under the normality assumption, we would have needed 16 participants per condition. Even though the data are nonnormal, they are only slightly skewed from normality, and this would only minimally increase the needed sample size for sufficient power. Median, IQR, and range for open-ended questionnaire scores for each document type are presented in Table 4.

### Number of Errors

The initial Kruskal-Wallis comparison of the Original and Dictionary conditions was insignificant (clinical trial: *P* = .20, Visit Notes: *P* = .91), so the two conditions were combined. The analysis found no significant differences, regardless of the document type. Median, IQR, and range for the number of errors for each document type are presented in Table 5.

**Table 5:** Total errors made

Document type and condition		N	Median	IQR^a^	Range	Contrast	*P* value
**Clinical trial**							
	Original	10	1.5	0.75–3.0	0–5	O^b^ vs D^c^	.99
	Dictionary	10	1.0	1.0–3.0	0–6		
	Combined (O^b^ + D^c^)	20	1.0	1.0–3.0	0–6	Kruskal-Wallis	.47
	Vocabulary	10	2.0	1.0–2.0	0–3		
	Coherent	10	2.0	1.0–3.25	0–6		
**Visit Notes**							
	Original	10	2.5	1.0–3.25	0–7	O^b^ vs D^c^	.91
	Dictionary	10	2.0	2.0–4.0	0–4		
	Combined (O^b^ + D^c^)	20	2.0	1.0–4.0	0–7	Kruskal-Wallis	.25
	Vocabulary	10	2.0	1.0–3.25	0–5		
	Coherent	10	3.5	2.0–5.0	0–10		

^a^ Interquartile range.

## Discussion

The results of this study expand our understanding of consumer difficulties with the technical language of medicine. Much research in this area has focused on terminology bridge solutions through technologies such as the Unified Medical Language System. Slaughter et al [36] looked at consonance of patient symptom expressions with nurses’ terminology in the medical record, but the goal of this research was to understand differences, not to measure incomprehension. Similarly, Hong et al [37] compared terminology in an electronic health record system with patient-friendly terms in the same system to find consonance between the two.

On a purely lexical basis, a translation from clinical to consumer language is appealing. Unfortunately, making complex clinical concepts clearer to laypeople requires more than a dictionary. The physician’s lack of time to explain concepts found in medical records was an often-cited criticism in the early literature concerning patient access to those records [8,11]. For this reason, two early studies built time and personnel resources into their design to avoid this problem. Golodetz et al [38] explained “necessary technical language” to the 60% of their study participants requesting this assistance (N = 103). Stein et al [39] provided their psychiatric patient participants with at least one nursing staff member to help explain terminology. Fischbach et al [40] surveyed the depth of the problem by designing a study in which patients and providers collaborated on authorship of the medical record: 20 patients with mixed diagnoses were asked to initiate and formulate their own problem list, with four providers suggesting modifications; both parties then worked together to write continuation notes (symptoms, clinical findings, and assessments). Fischbach et al found that physicians’ prospective worries about the time required to effectively communicate were entirely justified; these coauthoring consultations took as much as 50% longer than traditional visits; but these researchers saw value in incorporating the patient perspective into the health care documentation process. Participation in the coauthorship process “helped to eliminate serious misconceptions on the part of the patients.” In fact, a new language of cooperation was described as emerging out of this dialogue: “[T]he requirement for collaborative writing, which necessitated constant negotiation and feedback, created a meld of medical jargon and layman’s slang into a mutually useful language” (p 3). [40].

What do the results of this study tell us? We present here our original hypotheses:

H1: Readers’ comprehension of a text enhanced by providing standard, off-the-shelf dictionary definitions will not be significantly greater than their comprehension of the original text.

As expected, there was no difference in comprehension (as measured by the recall, answers to open-ended questions, or the number of errors) between the Original and Dictionary conditions. Comprehension was measured by the participants’ recall, their answers to open-ended questions, and the number of errors they made.

This supports the contention of Zeng and Tse [16] that the simple provision of a dictionary does not improve reader comprehension. However, it is important to remember that the dictionary is only a vehicle by which vocabulary is transported; vocabulary is the real problem, not the dictionary itself. Dictionary definitions may indeed be simple, clear, and likely to help the user; for the medical words we reviewed, however, the typical consumer dictionary was found to be extremely unuseful. For example, the National Library of Medicine’s consumer health website, MedlinePlus, is a portal intended explicitly for laypeople and not for health care professionals or researchers; among its licensed resources is the Merriam-Webster Medical Dictionary. This dictionary’s definition for acetaminophen reads as follows:

*a crystalline compound C* *8* *H* *9* *NO* *2* *that is a hydroxy derivative of acetanilide and is used in chemical synthesis and in medicine instead of aspirin to relieve pain and fever—called also* paracetamol*; see liquiprin, panadol, tylenol* [41]

Readers who does not know what acetaminophen is are unlikely to be assisted by this information.

H2: Readers’ comprehension of a vocabulary-enhanced text will be significantly greater than in the Original and Dictionary conditions combined.

This hypothesis was not supported. A specifically contextualized vocabulary developed for the purposes of this text did not improve comprehension, as assessed by any of the three comprehension measures. The lack of positive effect of a carefully constructed, clear vocabulary is counterintuitive. This result may be attributable to our choice of very complex medical texts for the study. The conceptual density of these texts may have created coherence gaps that were too large to be ameliorated by vocabulary definitions.

H3: Readers’ comprehension of a text with improved coherence will be significantly greater than in the Original plus Dictionary condition.

This hypothesis is partially supported, for the Visit Notes document but not for the clinical trial document. The *P* value for the Visit Notes in the Coherent condition compared with the Original plus Dictionary conditions is significant at .04. For this particular hypothesis, then, the researcher’s glass is half empty *and* half full. Many cognitive studies in other fields have shown that coherence is a factor affecting comprehension. Our results show that this is true for the Visit Notes document, a particularly impressive finding because, as discussed above, improving coherence of this document required making an already long text even longer—while the original Visit Notes document was 326 words long, the version with enhanced coherence totaled 1219 words. Despite this fourfold increase in length, the more coherent document still managed to hold the participants’ attention. Examples showing the difference between a participant with high recall in the Coherent condition and one with a low recall with the Original text condition appear in Textboxes 4 and 5. Each example is a description of the cardiac problems remembered from the Visit Notes.

For the clinical trial document, however, this is still not a promising result. While the median recall was increased from 9.0 to 10.5 propositions in the Coherent condition over the Original and Dictionary conditions, the error rate remained the same in the Coherent condition as in the other conditions; that is, no matter what was done to the text, the number of errors remained constant. The clinical trial document, then, was apparently simply so difficult, and so short, that nothing was able to make it easier to read.

H4: Readers’ comprehension of the Coherent condition will be significantly greater than in the Vocabulary condition.

This hypothesis was rendered irrelevant by the overall lack of significant comprehension improvement in the Vocabulary condition. Our expectation had been that both the Vocabulary and the Coherent conditions would improve comprehension compared with the Original and Dictionary conditions, with the gain being greater for the Coherent conditions. In this study, however, the improvement was observed only for the Coherent condition (and then only for the Visit Notes text).

**Excerpt of Visit Notes Text About Cardiac Problems Composed by Participant 28 Showing High Recall of Propositions [total of 43] in the Coherent Condition**

Heart:

1. The blood vessels are tightening as the result of a build up of cholesterol.

2. The patients heart beats irregularly

3. The patient has a pacemaker device to help control the hearts beat, this works by sending an electric pulse when the patients heart gets off beat.

4. There is a particular weakness in the left ventricle of the patient’s heart.

5. The patient is on blood thinners to reduce the risks of clotting which are a special threat for patients having suffered a heart attack, such as this patient.

**Excerpt of Visit Notes Text About Cardiac Problems Composed by Participant 5 Showing Very Low Recall of Propositions [total of 16] in the Original Text Condition**

Patient Visit

b) Irregular heartbeat, wheezing, strong carotid pulse, soft abdomen, good oral hygiene, heart murmur, supple neck

### Limitations

This study has limitations that may restrict the generalizability of its results. These include its small size (80 participants) and the educational background of the research participants: 90% were college graduates. This educational background, however, does allow us to make the suggestion that people with less education could have performed even more poorly. Additionally, our conclusions may be confounded by the fact that we tested only one clinical trial and only one Visit Notes document. It is difficult to say, for example, whether a clinical trial involving rheumatoid arthritis and Visit Notes involving pregnancy would have evoked different readability responses in our participants.

### Conclusion and Future Directions

We conclude by reviewing the findings of this study and examining their implications for future work. The practical significance of this study lies in showing the full extent of the difficulty and labor intensiveness of improving comprehension of clinical documents. This work explores cognitive characteristics of the reader–text match that show why commonly attempted solutions—lowering readability scores and providing dictionary definitions—are not sufficient. It also points to strategies for intervention that merit future research attention. Much research effort could be directed at (1) identifying aspects of coherence that are particularly relevant for comprehending complex medical texts, and (2) seeking automatic tools that can aid in document revision. Statistically, we have shown that improving coherence of typical clinical documents has a small effect on consumer comprehension, but this task is not scalable with automated solutions and would be impractical with manual solutions. Perhaps a promise of automation scalability lies in an iterative hybrid approach, where automated textual analysis for coherence is followed by manual editing, which is then rechecked with an automated tool. While automatic text editing is still a matter of the distance future, validated automated tools capable of distinguishing between high- and low-coherence versions of textual documents do exist [26]. Unfortunately, while an automated approach is well fitted for analyzing indices of local coherence, such as argument overlap, it is not capable of assessing many aspects of global coherence, such as the appropriateness of topic sentences and the background information level. In the case of knowledge-rich texts, such as medical documents, increasing local coherence alone is likely to be insufficient. Further research is needed using texts from more diverse clinical domains and more heterogeneous participants, including actual patients.

Second, it is interesting that the coherence-enhanced Visit Notes document was able to hold readers’ attention despite the fact that increasing coherence almost quadrupled the size of the document. This finding has implications not only for coherence, but also for text construction itself. It may be the narrative format that allows lay readers to form a more coherent story. Thinking of the medical record as narrative is a well-established trope in the medical humanities; Epstein, for example, writing about the development of genetics, points to the importance of the physician as writer: “a chronicler of bodily events and systematic narrator of particular phenomena in a particular context” [42]. Kennedy points to the “case...as the predominant form of medical narrative” and argues that it cannot be understood “aside from its involvement with literary discourse” [43] Recent work on illness narratives constructed from diaries—written by both nurses [44] and patients [45]—reveals that narrative structure assists participants in health care in sense-making—constructing a coherent account of the illness. In fact, considered in this light, the 30-year-old study by Fischbach et al [40] may have been as much about narrative as it was about medical record co-construction.

Finally, our results suggest that given the difficulty of engineering comprehensibility of clinical text, the most useful informatics tools will be those that can support the physicians, nurses, and patient educators tasked with making clinical information understandable to patients. These health care professionals use a repetitive cycle of explaining concepts, asking questions to ensure that patients comprehend, and explaining again. If the attainment of coherence is the end result of an iterative process, no single instance of a static document will solve the coherence problem.
